# Prevalence and risk factors of diabetic retinopathy among Chinese adults with type 2 diabetes in a suburb of Shanghai, China

**DOI:** 10.1371/journal.pone.0275617

**Published:** 2022-10-04

**Authors:** Huiling Tan, Xin Wang, Kaiyou Ye, Jianmin Lin, E. Song, Lihua Gong

**Affiliations:** 1 Department of Ophthalmology, Qingpu Branch of Zhongshan Hospital Affiliated to Fudan University, Shanghai, China; 2 Bengbu Medical College, Bengbu, Anhui, China; 3 Qingpu Center for Disease Prevention and Control, Shanghai, China; 4 Clinical Laboratory, Qingpu Branch of Zhongshan Hospital Affiliated to Fudan University, Shanghai, China; 5 Department of Ophthalmology, Lixiang Eye Hospital of Soochow University, Suzhou, Jiangsu, China; Indiana University Purdue University at Indianapolis, UNITED STATES

## Abstract

**Background:**

To investigate the prevalence and risk factors of diabetic retinopathy (DR) in a Chinese population with type 2 diabetes mellitus (T2DM) in a suburb (Qingpu) of Shanghai, China.

**Methods:**

A population-based cross-sectional study. A total of 7462 residents with T2DM in Qingpu were enrolled according to the resident health archives from January 2020 to December 2020. Blood and urine samples of the subjects were collected. Disc- and macula-centred retinal images were taken to assess DR. SPSS was used to analyse and investigate the prevalence and risk factors of DR.

**Results:**

The fundus images of 6380 (85.5%) subjects were of sufficiently good quality for grading. The average (range) age of 6380 subjects was 63.46±7.77 (28–92) years. Six hundred forty-four subjects were diagnosed with DR. The prevalence of DR was 10.1% (95% CI 9.4%-10.8%), with mild, moderate, and severe non-proliferative retinopathy and proliferative retinopathy being 2.1%, 6.3%, 1.3% and 0.4%, respectively. The prevalence of bilateral DR was 6.5%. Higher T2DM duration (OR, 1.057), fasting plasma glucose (OR, 1.063), glycated hemoglobinA1c (OR, 1.269), urea nitrogen (OR, 1.059), and urinary albumin (OR, 1.001) were associated with the higher DR prevalence.

**Conclusion:**

The prevalence of DR among Chinese adults with T2DM in Qingpu was 10.1%, in which non-proliferative DR was more common. Higher fasting plasma glucose and glycated hemoglobinA1c are well-known risk factors of DR, consistent with the findings in our study. Our study didn’t find the risk between lipid indicators and DR. However, several renal function indicators, like higher urea nitrogen and urinary albumin, were risk factors for DR in this study. Appropriate diagnosis and intervention should be taken in time to prevent and control DR development.

## Introduction

With the increasing ageing of the population, diabetes mellitus (DM) has become a global public health issue. It is estimated that there will be around 642 million DM patients in 2040 [1]. Diabetic retinopathy (DR) is a common microvascular complication of DM, which is the main reason for blindness and visual impairment in adults [2]. Although numerous studies have been conducted to report the prevalence and risk factors of DR, we are still short of data from low-income countries, which may not fully reveal the geographical and ethnic variability in the epidemiological characteristics of DR. About 75% of DM patients live in low-income countries [1], and China is the largest developing country in the world. The sample site that we chose is an underdeveloped region of Chinese megacities, which can well mirror the impact of urbanization on the development of DR. This information would provide references for similar studies in underdeveloped regions of megacities in China and many other developing countries.

Therefore, we performed this population-based study in a large T2DM cohort in suburban, southeast China. The purpose of this study is two-fold: First, to investigate the age- and sex-specific prevalence of DR in underdeveloped regions of megacities in developing countries and analyze the impact of urbanization on DR. Second, to examine the risk factors of DR in mainland China and better understand the relationship between DR and DM-related clinical indicators.

## Subjects/Materials and methods

### Study population

This was a population-based cross-sectional study. A total of 7462 permanent residents with T2DM in Qingpu, a suburb of Shanghai, were recruited according to the Qingpu Resident Health Archives (QRHA) from January 2020 to December 2020. The QRHA was a computerized database established in June 2000 at the Shanghai Disease Prevention and Control Center, which was set up with the aim of promoting the health management of residents. Data of the basic resident demographics (including name, gender, age etc.) were collected and could be shared in this database. Approximately 483 000 permanent residents are living in Qingpu, and 24322 residents are T2DM patients based on the QRHA. Seven thousand four hundred sixty-two residents with T2DM were identified using random cluster sampling from 12 administrative divisions of the Qingpu district. Researchers could identify participants during or after data collection. The study was approved by the Ethics Committee of Qingpu Branch of Zhongshan Hospital affiliated to Fudan University (registration number: #QP-2019-32) and complied with the Declaration of Helsinki. Written informed consent was obtained from each subject.

### Study procedure

All of the data were obtained from three parts: the QRHA, biochemical measurement and fundus images.

The information of name, gender, age, height and weight was acquired from the QRHA. Height and weight were measured by healthcare professionals using standardized measuring tools while the subjects were barefoot and were wearing as little clothing as possible. Body mass index (BMI) was calculated as weight divided by height squared (kg/m^2^).

Biochemical measurements, including blood analysis and urine analysis, were carried out to check fasting blood glucose (FBG), glycated hemoglobin A1c (HbA1c), TG, TC, HDL-C, low-density lipoprotein cholesterol (LDL-C), blood urea nitrogen (BUN), serum creatinine (Scr), urinary creatinine (Ucr) and urinary albumin (U-Alb). TC/TG ratio (Total Cholesterol to Triglyceride Ratio) and UACR were calculated by formulas as follows: TC/TG ratio = TC (mmol/L)/TG (mmol/L); UACR = U-Alb (mg/L)/Ucr (g/L). Blood samples and urine samples were both obtained under an overnight fasting condition and tested by the Clinical Laboratory of Qingpu branch of Zhongshan Hospital affiliated to Fudan University.

Disc- and macula-centered retinal images of both eyes of each subject were taken using the non-mydriatic digital fundus camera (Canon CR6-45NM, USA) by trained technicians. And the retinal images were stored and uploaded via the Ophthalmology PACS systems of Wonders Information Co. Ltd.

### DR grading procedure

A total of 6380 (85.5%) subjects’ fundus images were of good quality for grading. The interpretation of fundus images was made by the ophthalmologists from the Ophthalmology department of Qingpu branch of Zhongshan Hospital affiliated to Fudan University. The senior ophthalmologist conducted random checks to guarantee the diagnostic accuracy of the results. The diagnosis and grading of DR were made based on the International Clinical Diabetic Retinopathy Severity Scale [3]. A five-stage severity classification for DR is shown in Table 1. -The poorer DR grade of the two eyes represented the final DR diagnosis of the subject, which provided the foundation to divide subjects into five groups.

**Table 1:** The International Clinical Diabetic Retinopathy Severity Scale.

Diabetic Retinopathy Severity Level	Findings Observable upon Ophthalmoscopy
No diabetic retinopathy (NDR)	No abnormalities
Mild non-proliferative DR (Mild NPDR)	Microaneurysms only
Moderate NPDR	More than mild NPDR but less than severe NPDR
Severe NPDR	No signs of proliferative retinopathy (PDR) and any of the 4:2:1 rule: >20 intraretinal hemorrhages in each of 4 quadrants; definite venous beading in 2 quadrants; prominent intraretinal microvascular abnormalities in 1 quadrant.
Proliferative DR (PDR)	Neovascularization or vitreous/preretinal hemorrhage

### Statistical analysis

Microsoft Excel 2016 was used to input and process data. The mean and standard deviation ($\bar{\chi }\pm \text{s}$) was used to describe a normal distribution continuous data, and the median and interquartile ranges (medians (IQR)) were used to describe non-normal distribution continuous data. The percentage was used to describe categorical data. The prevalence of DR was analyzed by gender and age. Statistical analysis of risk factors for the development of DR was performed as follows. First, univariate analysis was used to screen out correlation factors. Mann–Whitney U test and analysis of variance were used to analyze normally distributed continuous data. A Kruskal–Wallis H test, Wilcoxon test and Chi-square were used for comparisons of categorical data, and non-normally distributed continuous data. Second, logistic regression models were applied to estimate the ORs and 95% CIs for all potential risk factors of DR. Third, principal component analysis (PCA) was used to filter out the most prevalent risk factors associated with the causation of DR in this cohort. Last, we did a Spearman correlation analysis to investigate the correlation between the most prevalent risk factors found in PCA with the intensity of fundus degeneration. The PCA and Spearman correlation analysis were performed in R-4.1.1. Other analyses were performed by SPSS 18.0 statistical software. A statistically significant difference was considered as p<0.05.

## Results

### Characteristics of the study population

A total of 6380 subjects who had gradable retinal fundus images were recruited in this study. There were 2875 (45.1%) males among the 6380 subjects. The mean age of the subjects was 63.84±7.53 years. The average body mass index (BMI) was 24.61±4.45 kg/m^2^. More characteristics of the blood and urine samples of the subjects are shown in Table 2.

**Table 2:** Characteristics of study subjects by the presence of DR.

Variables	All	NDR	Mild NPDR	Moderate NPDR	Severe NPDR	PDR	*P*
**Male gender**	2875	72	173	41	11	72	0.209**b**
**Age (years)**	63.84±7.53	63.94±7.49	61.41±8.40	63.38±7.65	62.26±8.00	64.85±5.45	**0.001b**
**Height (cm)**	161.56±7.60	161.59±7.57	161.94±7.73	160.86±8.04	162.41±7.83	160.74±6.52	0.185**b**
**Weight (kg)**	64.26±11.87	64.40±12.02	62.85±10.23	63.18±10.77	62.80±8.76	62.48±9.36	**0.026b**
**BMI (kg/m2)**	24.61±4.45	24.66±4.55	23.92±3.20	24.37±3.52	23.8±2.94	24.16±3.25	**0.009b**
**DM duration (years)**	6.40 (2.70–12.00)	6.00 (2.50–11.18)	8.00 (3.90–14.00)	9.00 (4.83–15.00)	8.00 (3.13–15.75)	13.00 (8.00–18.00)	**<0.001b**
**FPG (mmol/L)**	8.89±2.64	8.71±2.48	9.76±2.89	10.48±3.58	10.97±3.49	10.79±3.30	**<0.001a**
**HbA1c (%)**	7.30±1.47	7.20±1.42	7.88±1.51	8.14±1.73	8.34±1.74	8.37±1.76	**<0.001a**
**TG (mmol/L)**	1.37 (0.91–2.09)	1.39 (0.92–2.10)	1.27 (0.87–1.93)	1.26 (0.85–1.93)	1.28 (0.77–2.15)	1.44 (0.81–2.15)	0.101**b**
**TC (mmol/L)**	5.35±1.13	5.34±1.12	5.11±1.09	5.44±1.31	5.67±1.17	5.28±1.13	**0.004a**
**TC/TG ratio**	4.41±2.66	4.39±2.65	4.46±2.46	4.72±2.78	4.78±2.99	4.44±2.31	0.084**a**
**HDL-C(mmol/L)**	1.33±0.31	1.33±0.31	1.29±0.30	1.38±0.34	1.38±0.33	1.44±0.38	**0.007a**
**LDL-C(mmol/L)**	3.22±0.95	3.21±0.94	3.08±0.98	3.30±1.03	3.35±0.95	3.08±1.01	0.081**a**
**BUN (mmol/L)**	5.99±1.66	5.95±1.60	6.04±1.65	6.27±2.18	6.61±2.40	6.25±1.96	**0.009a**
**Scr (μmol/L)**	61.69±20.64	61.57±19.35	59.59±16.55	63.29±33.45	64.76±28.15	64.52±27.52	0.352**a**
**Ucr (μmol/L)**	18.00 (7.00–9284.00)	1466.50 (7.00–9666.75)	17.00 (8.00–8065.00)	8.00 (5.00–4516.25)	7.00 (4.00–17.25)	7.00 (4.00–4309.00)	**<0.001b**
**U-Alb (mg/L)**	12.30 (5.23–33.20)	12.10 (5.20–31.80)	10.00 (4.80–33.30)	15.00 (5.78–73.98)	23.90 (8.90–66.63)	11.20 (4.20–173.00)	**<0.001b**
**UACR (mg/g)**	12.10 (5.90–33.18)	11.70 (5.70–30.78)	10.70 (5.40–33.30)	19.15 (7.48–83.83)	33.15 (9.38–93.75)	20.30 (9.30–177.60)	**<0.001b**

NDR, no diabetic retinopathy; NPDR, non-proliferative diabetic retinopathy; PDR, proliferative diabetic retinopathy; BMI, body mass index; DM, diabetes mellitus; FPG, fasting plasma glucose; HbA1c, glycated hemoglobin A1c; TG, triglyceride; TC, total cholesterol; HDL-C, high-density lipoprotein cholesterol; LDL-C, low-density lipoprotein cholesterol; BUN, blood urea nitrogen; Scr, serum creatinine; Ucr, urinary creatinine; U-Alb, urinary albumin; UACR, urine albumin-to-creatinine ratio. Results are expressed as mean ± SD or medians (IQR); p values of DR group were compared by: a = analysis of variance, b = Kruskal–Wallis H test.

### Prevalence of DR in patients with T2DM

The age- and sex-specific prevalence of DR are presented in Table 3. The prevalence of DR did not differ statistically by gender, but the prevalence of any DR in objects with T2DM differed by age and peaked between 40 and 49 years of age. The estimated prevalence of any DR in patients with T2DM was 10.1% (644/6390, 95% CI 9.4% to 10.8%), and the prevalence of bilateral DR was 6.5%. The stage of mild and moderate NPDR were more common, and the total prevalence rate was 8.4%.

**Table 3:** Age-and gender-specific prevalence (95% CI) of DR in study subjects.

	N	Any DR	Mild NPDR	Moderate NPDR	Severe NPDR	PDR	Bilateral DR
**All**	6380	10.1(9.4–10.8)	2.1(1.8–2.5)	6.3(5.7–6.9)	1.3(1.0–1.5)	0.4(0.3–0.6)	6.5(5.9–7.1)
**Gender**							
Male	2875	10.3(9.2–11.4)	2.5(1.9–3.1)	6(5.1–6.9)	1.4(1.0–1.8)	0.4(0.2–0.6)	6.7(5.8–7.6)
Female	3505	9.9(8.9–10.9)	1.8(1.4–2.2)	6.5(5.7–7.4)	1.1(0.8–1.5)	0.5(0.3–0.7)	6.3(5.5–7.1)
*p*-value		0.570	0.051	0.399	0.263	0.651	0.547
**Age**							
≤39	22	9.1(0–22.2)	4.5(0.0–15.0)	4.5(0.0–15.0)	0.0(0.0–0.0)	0.0(0.0–0.0)	9.1(0.0–22.7)
40–49	198	16.7(11.7–21.7)	4.5(1.6–7.4)	9.1(5.3–13.1)	3.0(0.6–5.6)	0.0(0.0–0.0)	13.1(8.6–17.9)
50–59	1486	11.3(9.7–12.9)	2.8(2.0–3.7)	6.9(5.6–8.3)	1.3(0.8–1.9)	0.3(0.0–0.5)	7.9(6.6–9.3)
60–69	3216	10.0(8.9–11)	2.0(1.5–2.5)	6.0(5.2–6.9)	1.4(1.0–1.8)	0.6(0.3–0.9)	6.4(5.6–7.2)
70–79	1380	8.3(7–9.8)	1.3(0.8–1.9)	5.9(4.8–7.1)	0.7(0.3–1.2)	0.4(0.1–0.7)	4.2(3.2–5.3)
≥80	78	9(3.1–16.2)	1.3(0.0–4.3)	6.4(1.5–12.3)	1.3(0.0–4.6)	0.0(0.0–0.0)	2.6(0.0–6.8)
*p*-value		**0.004**	**0.011**	0.473	0.118	0.601	**<0.001**

### Risk factors of DR

The distribution of exposure variables in different stages of DR is set out in Table 2. Univariate analysis was used to screen out correlation factors. The results showed that age, weight, BMI, T2DM duration, FPG, HbA1c, TC, HDL-C, BUN, Ucr, U-Alb, and UACR were correlation factors accompanying the occurrence and development of DR.

Multivariate logistic regression analysis was performed with the correlation factors shown in the univariate analyses to confirm the risk factors of DR (Table 4). Higher DM duration (OR = 1.057, 95% CI 1.042–1.072; p<0.001), higher FPG (OR = 1.063, 95% CI 1.020–1.108; p = 0.004), higher HbA1c (OR = 1.269, 95% CI 1.176–1.369; p<0.001), higher BUN (OR = 1.059, 95% CI 1.010–1.111; p = 0.018), higher U-Alb (OR = 1.001, 95% CI 1.001–1.002; p<0.001) were risk factors for the occurrence and development of DR in T2DM patients.

**Table 4:** Risk factors of DR in the study.

Variables	Age-gender adjusted OR (95% CI)	*P*
**Age (per 10 years)**	0.823 (0.737–0.919)	**0.001**
**Weight (per 10 kg)**	0.873 (0.746–1.023)	0.092
**BMI (per kg/m2)**	0.999 (0.956–1.044)	0.967
**DM duration (per year)**	1.057 (1.042–1.072)	**<0.001**
**FPG (per mmol/L)**	1.063 (1.020–1.108)	**0.004**
**HbA1c (per 1%)**	1.269 (1.176–1.369)	**<0.001**
**TC (per mmol/L)**	0.911 (0.842–0.985)	**0.020**
**HDL-C (per mmol/L)**	1.225 (0.926–1.621)	0.155
**BUN (per mmol/L)**	1.059 (1.010–1.111)	**0.018**
**Ucr (per μmol/L)**	1.000 (1.000–1.000)	**<0.001**
**U-Alb (per mg/L)**	1.001 (1.001–1.002)	**<0.001**
**UACR (per mg/g)**	1.000 (1.000–1.000)	0.976

**Fig 1:**
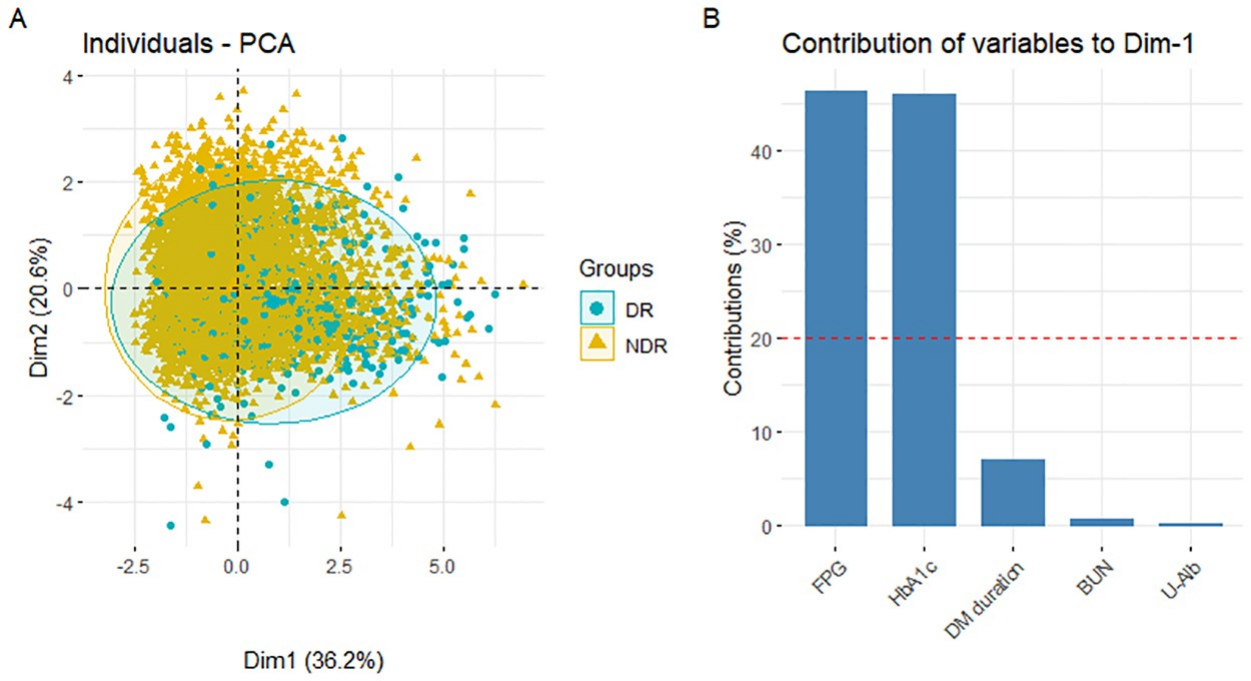
The results of principal component analysis (PCA). (A) The two-dimensional PCA results are based on the first and second principal components. (B) Contributions of variables to the first principal component. Abbreviations: DR, diabetic retinopathy; NDR, no diabetic retinopathy; FPG, fasting plasma glucose; HbA1c, glycated hemoglobin A1c; DM, diabetes mellitus; BUN, blood urea nitrogen; U-Alb, urinary albumin.

Next, for the risk factors screened out by multivariate logistic regression analysis, principal component analysis (PCA) was used to filter out the most prevalent risk factors associated with the causation of DR in this cohort. Results from PCA are shown in Fig 1. Fig 1A shows the two-dimensional PCA results based on the first principal component (Dim1) and second principal component (Dim2), which together explained 56.8% of the variance. FPG and HbA1c are the major contributions of variables to the first principal component.

**Fig 2:**
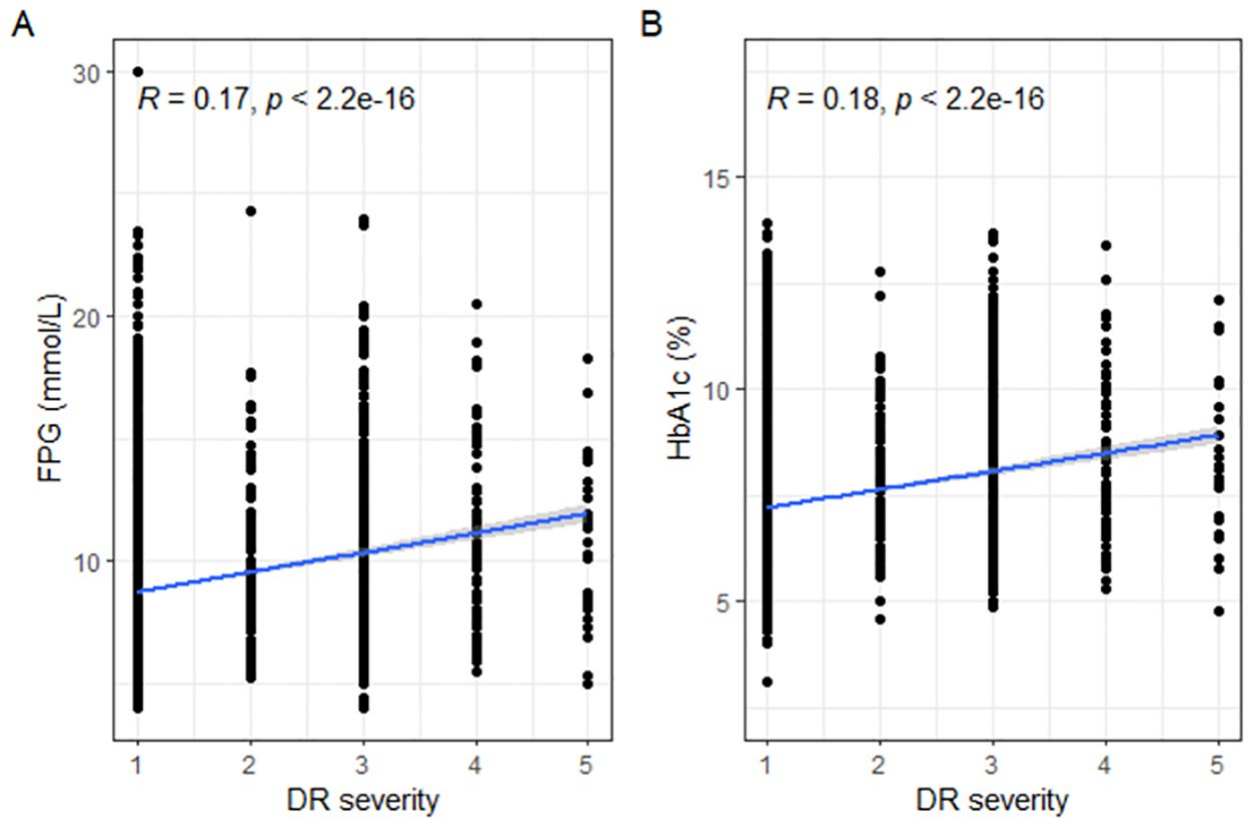
Spearman correlation analysis results about the correlation between FPG (A)/ HbA1c (B) and the intensity of fundus degeneration. R, correlation value by Spearman correlation analysis; P, significance level in Spearman correlation analysis. Abbreviations: DR, diabetic retinopathy; DR severity: 1(NDR, no diabetic retinopathy), 2(Mild NPDR, mild non-proliferative diabetic retinopathy), 3(Moderate NPDR), 4(Severe NPDR), 5(PDR, proliferative diabetic retinopathy); FPG, fasting plasma glucose; HbA1c, glycated hemoglobin A1c.

For the most prevalent risk factors found in PCA, we did a Spearman correlation analysis to investigate the correlation between them (FPG and HbA1c) with the intensity of fundus degeneration. Fig 2 shows the Spearman correlation analysis results. Fig 2A shows the positive correlation between FPG (p <0.001, r = 0.17) and the intensity of fundus degeneration. Fig 2B also showing the positive correlation between HbA1c (p <0.001, r = 0.18) and the intensity of fundus degeneration.

## Discussion

The prevalence of any DR, mild, moderate and severe NPDR, PDR in T2DM patients were 10.1%, 2.1%, 6.3%, 1.3%, and 0.4%, respectively in this study. Indicators reflecting glucose and lipid metabolism (higher fasting plasma glucose and glycated hemoglobinA1c) and renal function (higher urea nitrogen and urinary albumin) were risk factors of DR.

The prevalence of DR in the patients with T2DM identified in our study was 10.1%, which was lower than the global prevalence (25.16%) [4] and also lower than that of countries in other continents such as New Zealand (22.5%) [5], Uganda (19.5%) [6], Denmark (21.2%) [7], the UK (28.3%) [8], Canada (25.1%) [9]. The ethnic difference was the main factor leading to the prevalence differences among different continents. Asia, the continent that our study belonged to, remained the lowest DR prevalence [10]. The different underlying genetic and biological characteristics may explain the different prevalence in different ethnic populations. When compared to the results in other developing countries in Asia, the prevalence in Iran (37.8%) [11], Indonesian (43.1%) [12], India (32.53%) [13], Bangladesh (25.1%) [14] are higher than the present DR prevalence. Actually, the Chinese pooled DR prevalence (18.45%) [15] is significantly lower than in other countries and continents. The different prevalence may be correlated with socioeconomic status in developing countries. What’s more, regarding the prevalence of any DR at regional geographic level, it was reported that the prevalence of any DR in middle-aged and older Chinese with DM was the lowest in East China [15], which Shanghai is located.

The present DR prevalence was similar to the prevalence in some population-based studies conducted in Chinese mainland cities such as Liaoning (11.9%) [16], Weitang (10.38%) [17], Yangxi (8.19%) [18], Beijing (8.1%) [19] and communities in Shanghai (9.4%) [20]. Interestingly, the present DR prevalence was also lower than that in some other Chinese mainland cities such as Handan (14.24%) [21], Dongguan (18.2%) [22], and Shenzhen (18.58%) [23] and Suzhou (18.0%) [24]. Different sampling methods and characteristics of the study population may lead to different DR prevalence. The DR frequency of patients with a duration of DM of >10 years was about eight times that of <5 years [22]. In this study, 40.1% of patients with a duration of <5 years and the mean duration of T2DM is 6.40 (2.70–12.00) years, which is lower than the DM duration in Suzhou (10.5 ± 7.1 years) [24]. Moreover, studies with lower DR prevalence, such as Yangxi (8.19%) (18) and Beijing (8.1%) [19], are with more short duration or newly diagnosed T2DM participants.

As to other possible reasons for the relatively low DR prevalence in our study, the improvement in the control of modifiable risk factors might contribute to it. The mean HbA1c is 7.3% in our study vs 7.88% in a Singapore Study [25], which reflects relatively better physical conditions of participants in our study. Research in Northern Spain (8.56%) [26] also indicated that decreased DR prevalence was associated with the improvement of controlling risk factors. More discrepancies may have been introduced by the different types of DM in the study population and the cameras used to obtain the images.

We also observed that the prevalence of DR (10.1% in DM patients) in the current study (a suburb of Shanghai) was lower than that in urban areas of Shanghai. One recent study recruited T2DM patients from community health service centers in Shanghai and showed the prevalence of DR is 16.97% [27]. Another meta-analysis summarized six Shanghai DR prevalence studies [28], only one study was carried out in areas mixed urban and rural, and another five studies were carried out in the urban areas of Shanghai. Except a Shanghai diabetic complications study which contained pre-diabetic subjects, other Shanghai studies all showed higher DR prevalence (from 10.5% to 34.2%) than our study. Different to the urban areas of Shanghai, Qingpu, a suburb of Shanghai, is more like a representative of the underdeveloped regions of megacities. And the prevalence of DR in this study (10.1%) was similar to underdeveloped regions of other megacities in China such as Guangdong (8.19%) [18], Beijing (8.1%) [19]. The possible explanation of the low prevalence in underdeveloped regions of megacities could be as follows: On one hand, these regions have experienced urbanization and great economic development. A meta-analysis found that the prevalence of DR in the urban population was lower than that in the rural population in China [15], which indicated the importance of the economy. However, it also has been found that the prevalence of DM in urban residents was higher than that in rural residents, and urbanization was a factor that contributed to the increase in DM burden in China. Therefore, it could be that urbanization which associated with a sedentary lifestyle and a high-calorie diet was another factor that led to the increased prevalence of DM or DR. A research [29] also indicated that Indian immigrants living in a newly urbanized society had a higher prevalence of T2DM and DR than Indians living in urban India, largely stem from lifestyle changes. In this study, Qingpu was not a newly urbanised suburb. It had experienced urbanization for nearly 40 years and formed a stable lifestyle. Therefore, on the other hand, the retained healthy lifestyles of people such as low-calorie diet and high levels of physical activity in the underdeveloped regions of megacities could be another possible explanation of the low prevalence of DR.

Another factor that is attributed to the lower prevalence of DR in this study population may be the unique diet habits of the local population. There are three diet features in this study population according to the local CDC research [30], which belong to the "Jiangnan dietary pattern" [31]. First, Qingpu, as a suburb of Shanghai, had good compliance with the consumption of green leafy vegetables. The high vegetable and fish intake that may protect against the development of DR have been reported in two reviews [32, 33]. Second, the topography of Qingpu was a typical water system in the Yangtze River Delta, which led to the diet preference of eating freshwater fish instead of livestock as the main source of protein and fat intakes. The fat contained in livestock is mostly saturated fatty acids, while that in fish is mostly unsaturated fatty acids. It’s reported that increasing daily intake of polyunsaturated fatty acids (PUFAs) was associated with the reduced presence and severity of DR in well-controlled DM patients [34]. What should be noted is the protective effects of fish on DR didn’t just stem from the presence of PUFAs alone, and the exact whole mechanisms need to be further confirmed [33]. Third, Qingpu residents had a habit of long-term tea consumption. According to a Cross-sectional study, Shanghai had the highest tea consumption among four big Chinese cities [35]. In addition, a survey among elderly Chinese from rural communities of Suzhou reported that long-term tea consumption is associated with a reduced risk of DR [17]. The interesting point is that Qingpu is geographically adjacent to Suzhou and was a county of Suzhou before the year 1958. Moreover, the characteristics of the population in those two studies were similar (both suburban elderly populations), and the DR prevalence of Suzhou (10.38%) and Qingpu (10.1%) were very close and both in a low level. Therefore, the lower prevalence of DR in those two populations may be attributed to the diet habit of long-term tea consumption in the local elder adults. More relationships between diet and DR prevalence deserve to be further researched in later studies. For example, it appeared that foods containing vitamin D were beneficial to the improvement of DR [36]. Although Vitamin D deficiency was common in China, Vitamin D levels in suburban elderly populations of Shanghai were higher than that in urban has been reported in a cross-sectional study [37], consistent with the view in this study that the DR prevalence in rural areas was lower than that in urban areas of Shanghai.

FPG and HbA1c are the most prevalent risk factors associated with the causation of DR in this cohort, which is both positively correlated with the intensity of fundus degeneration. Chronic hyperglycaemia (reflected by DM duration and HbA1c) is also considered the major determinant for DR in the previous study [38]. Age was related to the prevalence of DR in our analysis: the prevalence of any DR increased with decreased age and peaked between 40 and 49 years of age. In a cohort study in the UK [8], the risk of developing DR peaked in those aged 55–64. And the DR prevalence was found to peak in the age group of 60–69 years in a meta-analysis [15]. Although the specific age reported by different studies was not the same, it was confirmed in the previous investigations and synthesized analyses that the DR prevalence in the elderly is lower than that in younger patients with DM. A meta-analysis [15] indicated that it was the combination of improved mortality and reduced DR incidence that drove the low prevalence in the elderly.

No clear association between TG or TC to DR has been established in previous studies. A multi-hospital-based cross-sectional study in China [39] indicated lower TG was associated with the presence of DR. Lower TC was associated with higher DR prevalence in the Singapore Eye Diseases Study [25]. In other population-based studies [7, 15, 16, 19, 20, 22, 40], neither TG nor TC was significantly associated with DR prevalence. Similar to the Singapore study, the present study suggests that TG is not associated with DR, while lower TC is associated with higher DR prevalence. Furthermore, this cross-sectional study revealed that there was no association between the TC/TG ratio and DR. The relationship between the TC/TG ratio and any measure of DR in the literature is limited. Only a Community-Based Study [41] among Chinese with T2DM reported a positive association between TC/TG ratio and DR prevalence, while the association is not observed in the current study. However, the TC/TG ratio was found to be associated with some metabolic factors that may contribute to DR. For example, a negative correlation was observed between the TC/TG ratio and the presence of LDL [42]. Moreover, the CALLISTO study [43] found the TC/TG ratio was significantly correlated with high-sensitivity C-reactive protein levels that can predict cardiovascular disease. Another study [44] reported the TC/TG ratio might play an important role in telomere length-related gestational DM risk and pathogenesis.

DR and diabetic nephropathy are both microvascular complications of DM, and the abnormal renal function was correlated with the development of DR [45]. Several renal function indicators, including BUN and U-Alb, are associated with the development of DR in patients with T2DM in this study. Although BUN was a risk factor for DR in this study, the risk of DR was not altered by BUN in another study [46]. However, in a multi-hospital-based cross-sectional study of 16 305 T2DM patients, BUN was an independent risk factor of DR [39]. UACR was often used to describe the U-Alb excretion, which could predict the risk of DR development and progression in T2DM patients [45, 47, 48]. Although UACR was not a risk factor for DR in the multivariate logistic regression analysis of this study, we found that patients with severe NPDR and PDR had a higher baseline UACR level as compared with those with NDR, mild and moderate DR. What’s more, higher U-Alb were found associated with higher DR prevalence in the current study, consistent with the relationship between DR and UACR (UACR = U-Alb/ Ucr).

The underlying mechanisms for the effects of risk factors on DR have been investigated in previous studies. Consistent with our study, chronic hyperglycaemia (reflected by DM duration and HbA1c) is the major determinant for DR [38]. One recent study found that high-glucose can induce retinal pigment epithelium mitochondrial pathways of apoptosis and inhibits mitophagy [49]. In addition, hyperglycaemia can induce intracellular excess glucose flux, overproduce reactive oxygen species, and increase the formation of AGEs, which lead to persistent damage to the retina [50].

Dyslipidemia pathophysiology is another mechanism impairing the retina [51]. The possible mechanism is that the metabolic dysregulation induced by dyslipidemia can promote the vasculature developing in the neural retina [52]. Moreover, clinical studies have proved that hyperglycaemia and dyslipidemia can impair the retina synergistically or additively. For example, glucose-mediated de novo lipogenesis in photoreceptors can drive early DR [53].

The kidney and the eye are an ’unhappy alliance’. DR and diabetic nephropathy have many risk factors in common. Systemic inflammation (reflected by albuminuria) induced by the damaged kidney is considered to accelerate the progression of both the eye and the kidney complications [54].

This study had some strengths. First, the sample size was large. Second, subjects were selected from the QRHA instead of hospitals, avoiding the selection bias. Third, the objects in this research could be representative of the urbanized suburban T2DM population in the economically developed areas of developing countries. Nevertheless, there were some limitations. The biggest limitation of this study was the data on blood pressure, and other potential risk factors of DR were not collected here because the related data in QRHA were incomplete. And this study lacked diet and physical activity data to clarify further the underlying mechanism of the low prevalence of DR. What’s more, optical coherence tomography could not be used to estimate DR and diabetic macular edema accurately. Finally, the impact of cataracts and other eye diseases on the assessment of fundus photos was difficult to avoid.

In conclusion, the current study provided new data on DR among Chinese adults with T2DM in the underdeveloped regions of megacities in China. The prevalence of DR was 10.1%, in which NPDR was more common. The lower prevalence of DR in the underdeveloped region of megacity may be attributed to the great economic development in urbanization, the retained healthy lifestyles, and the "Jiangnan dietary pattern". Indicators reflecting glucose and lipid profile as well as renal function were considered as risk factors of DR and discussed in this study. FPG and HbA1c are well-known risk factors for DR, consistent with the findings of our study. The relationship between lipid indicators and DR is complicated and not consistent in different studies. In our study, TG was not associated with DR, while lower TC was associated with higher DR prevalence. Several renal function indicators, like BUN and U-Alb, were risk factors for DR in this study. Longitudinal data are needed to investigate further the relationship of lipid and renal function indicators to DR.

## Supporting information

S1 File (XLS)
